# Analysis of intestinal microorganisms and metabolite in childhood allergic asthma: role in assessing the severity of condition in children

**DOI:** 10.3389/fped.2025.1680620

**Published:** 2025-12-11

**Authors:** Zhoubin Xu, Haichao Ma, Yujuan Liu, Shiming Liang, Zhongnan Liao

**Affiliations:** Department of Pediatrics, Shenzhen University General Hospital, Shenzhen, Guangdong, China

**Keywords:** childhood allergic asthma, intestinal microorganisms, metabolites, bifidobacteria, lactobacillus

## Abstract

**Background:**

Childhood allergic asthma is a widespread chronic respiratory condition that is becoming more common worldwide. Presently, the evaluation of its severity depends on clinical symptoms and inflammatory indicators like neutrophils and CRP, which are not very specific. Increasing research indicates that the gut microbiota and its metabolites—such as Bifidobacterium, Lactobacillus, short-chain fatty acids, and lipopolysaccharides—are important in immune system regulation and might affect asthma severity through the gut–lung connection. This study aims to determine if these microbial and metabolic factors can be used as new biomarkers to assess the severity of allergic in children.

**Methods:**

A retrospective analysis was conducted on the medical records of 148 pediatric patients diagnosed with allergic asthma who were admitted to our hospital between May 2023 and September 2024. The patients were categorized into mild-to-moderate and severe-to-critical groups according to established severity grading criteria during the acute exacerbation phase. Metabolite indices of intestinal microbiota were compared between the two groups, and correlation scatter plots were generated to examine the association between these metabolites and disease severity. Subsequently, a receiver operating characteristic (ROC) curve was constructed to evaluate the predictive value of intestinal microbiota metabolites for assessing the severity of allergic asthma in children.

**Results:**

No significant differences in neutrophil/leukocyte counts, CRP, or Klebsiella prevalence were observed between the mild-moderate and severe-critical pediatric allergic asthma groups (*P* > 0.05). However, the mild-moderate group showed significantly higher levels of Bifidobacteria, Lactobacillus, acetic acid, propionic acid, and butyric acid, but lower Escherichia coli and lipopolysaccharide (LPS) than the severe-critical group (*P* < 0.05). Disease severity negatively correlated with Bifidobacteria, Lactobacillus, and these three short-chain fatty acids, and positively with E. coli and LPS (*P* < 0.05). For predicting severity, the area under the curve (AUC) was 0.686 for Bifidobacteria, 0.785 for Lactobacillus, 0.811 for E. coli, 0.711 for acetic acid, 0.653 for propionic acid, 0.788 for butyric acid, and 0.671 for LPS. Notably, a combined model integrating these markers achieved an AUC of 0.956, significantly outperforming any single predictor (*P* < 0.05). These results indicate that gut microbiota-derived metabolites hold substantial potential as biomarkers for assessing disease severity in children with allergic asthma.

**Conclusion:**

The composition of intestinal microbiota and their metabolites exhibits abnormal expression patterns in children diagnosed with allergic asthma, correlating with the severity of the disease. These alterations may serve as significant biomarkers for predicting the clinical severity in pediatric patients with allergic asthma.

## Introduction

Allergic asthma is a chronic inflammatory disorder of the airways precipitated by allergen sensitivity, predominantly affecting children and representing a prevalent respiratory condition within pediatric populations. Epidemiological studies have consistently demonstrated a rising trend in the prevalence of childhood asthma over recent years, with a global average prevalence reported at 11.50% among children aged 6–7 years and 14.10% among those aged 13–14 years (1, 2). Furthermore, allergic asthma has been identified as a major phenotype of pediatric asthma, constituting over 80% of cases in this demographic (3). In recent years, the rapid urbanization and the accelerated development of infrastructure have contributed to significant air pollution. This environmental degradation, combined with the underdeveloped immune systems of children, has consequently heightened the risk of allergic asthma among the pediatric population (4). Allergic asthma frequently manifests during nighttime or early morning hours. If not promptly identified and managed with appropriate interventions, it can readily lead to bronchial obstruction, thereby facilitating the progressive worsening of the disease. This is particularly concerning in cases of severe and critical allergic asthma, which can significantly impair the quality of life in pediatric patients (5). Consequently, early evaluation of disease severity is essential for effective clinical management.

Currently, the clinical assessment of the severity of allergic asthma in pediatric patients primarily relies on the evaluation of clinical symptoms and inflammatory response-related biomarkers. Indicators such as neutrophils, leukocytes, and C-reactive protein (CRP) hold significant clinical relevance in the diagnosis and severity assessment of allergic asthma. However, there remains a deficiency in specific and practical diagnostic markers that can be effectively utilized in clinical practice (6). With the ongoing advancement of clinical research, it has been increasingly recognized that intestinal microorganisms play crucial physiological roles in gastrointestinal diseases and the maintenance of immune homeostasis. Furthermore, these microorganisms can modulate the body's immune functions through their metabolites, thereby contributing to the preservation of immune balance (7). A prior investigation suggested that the pathogenesis of asthma is associated with the excessive activation of Th2-type immune responses, which may be modulated by changes in the intestinal microbiota via multiple mechanisms. This finding indicates a complex relationship between allergic asthma and the composition of intestinal microorganisms (8). Bifidobacteria, Lactobacillus, Escherichia coli and Klebsiella are prevalent intestinal microorganisms that play a crucial role in maintaining intestinal health, modulating the immune system, and facilitating nutrient absorption. Bifidobacteria represent a critical group of probiotics within the intestinal microbiota, possessing the capacity to modulate the Th1/Th2 immune balance, thereby correcting immune dysfunctions observed in pediatric asthma patients and enhancing immune responses within pulmonary tissues. Similarly, Lactobacillus constitutes a significant probiotic genus that produces short-chain fatty acids, which play a pivotal role in regulating the intestinal microbiome, mitigating inflammatory processes, and modulating both the gut flora and immune system to alleviate symptoms of allergic asthma. In contrast, Escherichia coli, a acultative pathogenic bacterium commonly colonizing the intestinal tract, can, upon excessive proliferation, disrupt microbial homeostasis and substantially elevate the risk of allergic asthma development. Additionally, Klebsiella, a facultative anaerobic Gram-negative bacterium, is implicated in dysbiosis through its abnormal overgrowth, exerting considerable influence on immune system function (9, 10). Furthermore, short-chain fatty acids and lipopolysaccharides are metabolic products derived from intestinal microbiota. Short-chain fatty acids, such as acetic acid, propionic acid, and butyric acid, possess significant physiological functions and are critically involved in the regulation of immune responses and the modulation of inflammatory processes. These activities influence the pathogenesis of asthma. Lipopolysaccharide, a constituent of the outer membrane of Gram-negative bacteria, exhibits immunostimulatory properties. Its levels are elevated in asthma, a phenomenon associated with dysbiosis of the intestinal microbiota and an increase in opportunistic pathogenic bacteria (11).

To date, the majority of research investigating metabolites in allergic asthma has concentrated on biological samples such as blood and urine. However, the association between metabolites within the intestinal tract—the primary habitat of the microbiota—and asthma remains unexplored. This study systematically examines the role of intestinal microbial metabolites in evaluating pediatric allergic asthma, aiming to quantify the correlation between metabolite levels and disease severity, thereby establishing a biomarker foundation for clinical classification.

## Methods

### Research object

A retrospective analysis was conducted on the medical records of 148 pediatric patients diagnosed with allergic asthma who were admitted to our hospital between May 2023 and September 2024. These patients were classified into mild-moderate and severe-critical groups based on established criteria for the severity of acute exacerbations. The study received approval from the Medical Ethics Committee, complied with national laws and regulations, and was conducted in strict accordance with the relevant provisions of the *Declaration of Helsinki of the World Medical Association*.

### Inclusion and exclusion criteria

Inclusion criteria: (1) fulfillment of the diagnostic standards for pediatric allergic asthma (12); (2) presentation during the acute exacerbation phase; (3) availability of comprehensive medical records and pertinent examination data for the pediatric patients; (4) patients being treatment-naïve prior to admission; and (5) absence of genetic disorders.

Exclusion criteria: (1) primary immunodeficiency disorders; (2) presence of pulmonary developmental anomalies, active tuberculosis, or other respiratory pathologies; (3) prior administration of probiotics, immunomodulatory agents, or glucocorticoids before admission; (4) history of major pulmonary surgery; and (5) existence of severe infectious or communicable diseases.

### General situation

Medical records of pediatric patients who satisfied the specified inclusion and exclusion criteria were obtained. These records encompassed data on gender, age, body mass index (BMI), monthly household income, residential location, mode of delivery, exposure to passive smoking, educational attainment of family members, duration of illness, precipitating factors, presence of comorbid allergic rhinitis, and familial history of allergic conditions.

### Grouping method

All pediatric subjects were classified into two groups mild to moderate and severe to critical based on established criteria for assessing the severity of acute exacerbations. The mild category was characterized by mild exertional dyspnea, a peak expiratory flow rate (PEF) exceeding 80% following β-agonist administration, normal arterial oxygen partial pressure (PaO2), arterial carbon dioxide partial pressure (PaCO2) below 45 mmHg, blood oxygen saturation (SaO2) above 90%, and normal blood pH levels. The moderate category included patients exhibiting dyspnea with minimal exertion, PEF ranging from 60% to 80% post β-agonist treatment, PaO2 between 60 and 80 mmHg, PaCO2 at or below 45 mmHg, SaO2 between 91% and 95%, and normal pH. Severe cases were defined by dyspnea at rest, PEF less than 60% after β-agonist use, PaO2 below 60 mmHg, PaCO2 exceeding 45 mmHg, SaO2 under 90%, and acidemia. The critical group was characterized by an inability to speak, pronounced dyspnea, PaO2 less than 60 mmHg, PaCO2 above 45 mmHg, SaO2 below 90%, and decreased pH levels (13).

### Intestinal microorganism metabolites

#### Intestinal microorganisms testing

**Sample Processing and DNA Extraction:** A 1-gram fecal sample from children was collected in a sterile, dry container. Subsequently, 9 mL of phosphate-buffered saline (PBS) was added, and the mixture was thoroughly agitated before centrifugation at 1,500 rpm (radius: 8 cm) for 5 min. This centrifugation step was repeated three times. The resulting supernatant was then subjected to centrifugation 9,000 rpm for 3 min. The pellet was washed three times with PBS. Finally, DNA extraction was performed using distilled water and polyethylene glycol octyl phenyl ether solutions, respectively.

**Quantitative real-time Polymerase Chain Reaction (qPCR) Detection:** qPCR was employed for the targeting of intestinal microorganisms, specifically Bifidobacteria, Lactobacillus, Escherichia coli and Klebsiella species. Fragments of the 16S rRNA gene from the target bacteria were cloned into the pMD19-T vector and subsequently transformed into competent Escherichia coli DH5α cells. Following the selection of positive clones, plasmid DNA was extracted. The concentration of the isolated plasmids was quantified using the Quant-it™ dsDNA HS reagent, and the plasmid copy number was determined based on the molecular weight of the plasmid. The plasmids underwent ten-fold serial dilutions to function as standards for the generation of a standard curve. For each dilution level, three replicate wells were prepared. The standard curve was required to exhibit a correlation coefficient (R²) of at least 0.99, with amplification efficiency maintained within the range of 90% to 110%. Universal primers were developed targeting the highly conserved region of the 16S rRNA gene. The sequences of the primers are as follows: forward primer is 5′-TCGTCGGCAGCGTCAGATGTGTATAAGAGAGACAGCCTACGCTACGGGNGGCWGCAG−3′, and the reverse primer is 5′-GTCTCGTGGGCTCGGAGATGTGTGTATAAGAGACAGGACTACHVGGGTATCTAATCC-3′. The reaction conditions consisted of an initial pre-denaturation step at 100 ℃ for 45 s, followed by denaturation at 95 ℃ for 5 s, annealing at 60 ℃ for 30s, and extension at 72 ℃ for 10 min. These steps were repeated for a total of 40 cycles. Subsequently, the PCR products were quantified using the Quant-iT™ dsDNA HS reagent. The quantity of the target bacterial copies within the samples was determined using the standard curve and subsequently converted into the absolute abundance of the target bacteria per gram of fecal material, with adjustments made for dilution encountered factors during sample processing.

**16s RNA Sequencing:** DNA products derived from multiple samples were combined and subsequently purified utilizing a suitable purification kit to eliminate impurities and short DNA fragments, thereby enhancing the quality of sequencing. The purified samples underwent high-throughput sequencing analysis utilizing the Illumina HiSeq 2,500 platform, provided by Biomarker Technologies Corporation in Beijing, China, to generate extensive 16S rRNA gene sequence datasets. The bacterial 16S rRNA gene sequences were retrieved and analyzed utilizing the Silva database to facilitate species identification and taxonomic classification based on sequence similarity. This approach enabled the determination of microbial community composition and the relative abundance of taxa within the samples.

#### Metabolite detection

**Short-chain fatty acids:** Fecal samples weighing 50 mg from children were collected, to which 1 mL of sodium hydroxide aqueous solution was added. This mixture was vortexed for 60 s and subsequently centrifuged at a speed of 1,500 rpm. After a 20 min incubation, the supernatant was collected and mixed with 300 µL of distilled water, followed by vortexing for 30s. Subsequently, 100 µL of n-propyl chloroformate and 500 µL of a pyridine propyl alcohol solution were added for derivatization. The mixture was vortexed for 60 s, then subjected to extraction n-hexane and centrifuged at 3,000 rpm for 5 min, retaining 200 µL of the extract. An additional 200 µL of n-hexane was added to the derivatives, and after extraction, 200 µL of the extract was preserved. The two extracts were combined and centrifuged again at 3,000 rpm for 5 min. Finally, gas chromatography-mass spectrometry (GC-MS) was employed to analyze the chromatograms and mass spectra of the short-chain fatty acids.

**GC-MS Detection Conditions:** An Agilent 7890A-5975C gas chromatography-mass spectrometry system equipped with a DB-FFAP capillary column was employed for the analysis. The initial oven temperature was maintained at 40 °C for a duration of 3 min, after which it was elevated to 150 °C at a rate of 5 °C/min and held constant for 2 min. Subsequently, the temperature was further increased to 230 °C at a rate of 10 °C/min and maintained for 5 min. The injector temperature was set at 250 °C, operating with a split ratio of 10:1. An injection volume of 5 μL was utilized, with high-purity helium serving as the carrier gas at a flow rate of 1.0 mL/min. The mass spectrometry parameters were configured as follows: an electron impact ionization operating at an ionization energy of 70 eV, an ion source temperature maintained at 230 °C, and a quadrupole temperature set to 150 °C. A solvent delay period of 3 min was implemented. Qualitative analysis was conducted using full-scan mode, whereas quantitative analysis employed selected ion monitoring mode.

**Quality Control:** (1) During the sample preparation process, 2-ethylbutyric acid was incorporated as an internal standard to account for variability in extraction efficiency and instrumental detection errors. The peak area ratios of acetic acid, propionic acid, and butyric acid relative to the internal standard were determined, and quantification was conducted using the established calibration curve. (2) Standard solutions of acetic acid, propionic acid, and butyric acid (concentration range: 0.1–100 μg/mL) were individually prepared. Subsequently, an internal standard was added, and derivatization was carried out. GC-MS analysis was then performed using the identical sample preparation procedure as previously described. The calibration curve was generated by plotting the concentrations of the standards along the *x*-axis against the corresponding peak area ratios of the standards to the internal standard on the *y*-axis. Each concentration level was analyzed in triplicate, and the calibration curve was considered acceptable only if the correlation coefficient (R²) was equal to or greater than 0.99. (3) For each batch of sample analyses, a blank control was incorporated to assess potential contamination from reagents. In instances where target short-chain fatty acids were identified in the blank control, the sample processing and analytical procedures were repeated.

**Lipopolysaccharide: A** fecal sample weighing 1 gram was collected from each child using a sterile, dry container. Phosphate-buffered saline (PBS) was added to the sample, which was then thoroughly mixed and centrifuged at 3,000 rpm for 10 min. Following centrifugation, the supernatant was carefully separated. The concentration of lipopolysaccharide level was quantitatively measured in strict accordance with the protocols specified by the enzyme-linked immunosorbent assay (ELISA) and the manufacturer's instructions of the kit (Wuhan Elite Bio-technology). **Quality Control:** (1) The correlation coefficient of determination (R²) for the standard curve is required to be at least 0.99. The absorbance measurement of the blank well should not exceed 0.1, and the absorbance values of the standard wells must demonstrate a distinct incremental trend corresponding to increasing concentrations. (2) For every batch of analyses conducted, quality control samples must be incorporated. The recovery rates of these quality control samples are required to lie within the interval of 85%–115% to guarantee the accuracy and reliability of the analytical results.

#### Laboratory tests

On the day of examination day, a 3 mL fasting venous blood sample was collected from each child and transferred into EDTA containing vacuum blood collection tubes. CRP levels were measured using the ADVIA2400 biochemical analyzer (Siemens, Germany), while neutrophil and leukocyte counts were determined utilizing the BC-6900 automated hematology analyzer (Myriad).

#### Observation on indicators

A total of 148 pediatric patients diagnosed with allergic asthma were categorized into mild-moderate and severe-critical groups according to established severity grading criteria during the acute exacerbation phase. Utilizing the intestinal microbial metabolite profiles from both groups, correlation scatter plots were generated to examine the associations between these metabolites and disease severity in the children. Furthermore, receiver operating characteristic (ROC) curve analyses were performed to evaluate the predictive utility of intestinal microbial metabolites for assessing the severity of the condition in this population.

#### Statistical analysis

In this study, data analysis was conducted using SPSS 25.0. Categorical variables were presented as frequencies and percentages[*n*(%)], and comparisons were made using the *χ*^2^ test. Continuous variables were assessed for normality using the Shapiro–Wilk test. Variables conforming to a normal distribution were expressed as mean ± standard deviation, with between-group comparisons performed using the independent samples *t* test, and within-groups comparisons conducted using the paired samples *t* test; Variables not conforming to normal distribution were reported as median with interquartile range [M (P_25_, P_75_)], and analyzed using the non-parametric Mann–Whitney *U* test. *P* < 0.05 was considered indicative of statistical significance.

## Results

### Comparison of medical records in the mild-moderate and severe-critical groups

A total of 148 pediatric patients diagnosed with allergic asthma were categorized into mild-moderate and severe-critical groups based on established severity grading criteria during the acute exacerbations. Specifically, the cohort comprised 69 mild cases, 44 moderate cases, 21 severe cases, and 14 critically severe cases, resulting in 113 patients classified within the mild-moderate group and 35 patients within the severe-critical group. A comparison of medical record data between the two groups indicated no significant differences in age, with the mild-moderate group averaging (7.97 ± 2.45 years) and the severe-critical group averaging(8.16 ± 2.32 years). BMI values were (19.42 ± 2.06) for the mild-moderate group and (18.75 ± 1.94) for the severe-critical group, with no statistically significant difference observed. Additionally, the mean monthly household income was comparable between the mild-moderate group (5,436.28) and the severe-critical group (5,451.39). Furthermore, disease duration did not differ significantly between the mild-moderate group (17.12 ± 4.56 months) and the severe-critical group (16.85 ± 4.15 months). No significant differences were observed between the mild-moderate and severe-critical groups regarding the prevalence of a history of passive smoking, coexisting allergic rhinitis, and family history of allergic diseases. Additionally, comparisons between these groups revealed no statistically significant differences in terms of gender, place of residence, mode of delivery, educational attainment of family members, and exposure to triggers (*P* > 0.05, Table 1).

**Table 1:** Comparison of medical records in the mild-moderate and severe-critical groups (*n* = 148).

Medical Records		Mild-moderate group (*n* = 113)	Severe-critical group (*n* = 35)	*χ^2^*/*t*	*P*
Gender	Male	68	22	0.081	0.777
	Female	45	13		
Age (years)		7.97 ± 2.45	8.16 ± 2.32	0.406	0.685
BMI (kg/m^2^)		19.42 ± 2.06	18.75 ± 1.94	1.704	0.091
Monthly household income (yuan/month)		5,436.28 ± 41.39	5,451.39 ± 44.06	1.859	0.065
Place of residence	Town	71	20	0.365	0.546
	Countryside	42	15		
Birth method	Cesarean	23	8	0.101	0.750
	Natural childbirth	90	27		
History of passive smoking	Yes	36	14	0.792	0.374
	No	77	21		
Literacy level of family members	Junior high school and below	28	10	0.201	0.654
	High school and above	85	25		
Disease duration (months)		17.12 ± 4.56	16.85 ± 4.15	0.312	0.755
Triggers	Dust mite allergy	46	17	1.255	0.534
	Inhalation of dust/pollen	23	8		
	Ingestion of fish and shrimp	44	10		
Combined allergic rhinitis	Yes	38	14	0.476	0.490
	No	75	21		
Family history of allergic diseases	Yes	24	11	1.537	0.215
	No	89	24		

### Comparison of laboratory indicators in the mild-moderate and severe-critical groups

**Figure 1:**
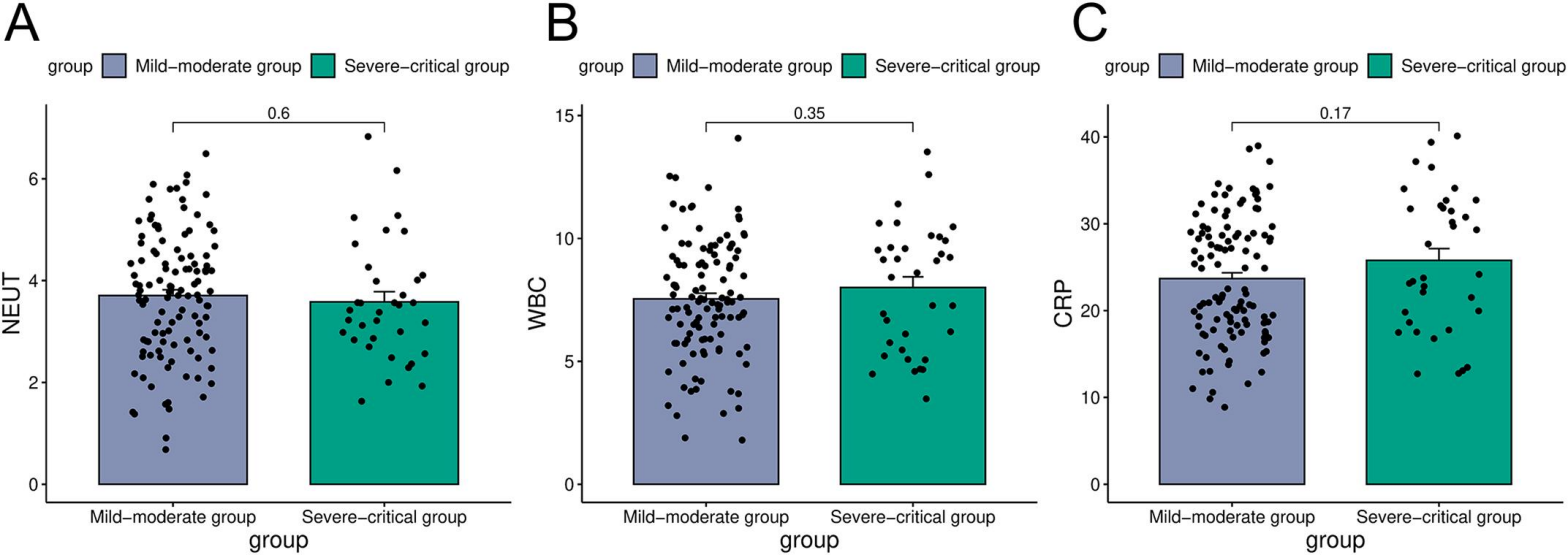
Comparison of laboratory indicators in the mild-moderate and severe-critical groups [**(A)** was the comparison of neutrophil counts in the mild-moderate and severe-critical groups; **(B)** was the comparison of white blood cell counts in two groups; **(C)** was the comparison of CRP in two groups].

The observational findings regarding laboratory indices in the mild-moderate and severe-critical groups indicated that the neutrophil count in the mild-moderate group was 3.7  ×  10^9^/L 1, which did not significantly differ from the 3.58 × 10^9^/L observed in the severe-critical group. Similarly, the leukocyte count in the mild-moderate group, measured at 7.54 × 10^9^/L, showed no significant difference compared to 8.01 × 10^9^/L in the severe-critical group. Furthermore, CRP levels were comparable between the two groups, with values of 23.68 × 10^9^/L in the mild-moderate group and 25.79 × 10^9^/L in the severe-critical group (*P* > 0.05, Figure 1).

### Comparison of intestinal microorganism indicators in the mild-moderate and severe-critical groups

**Figure 2:**
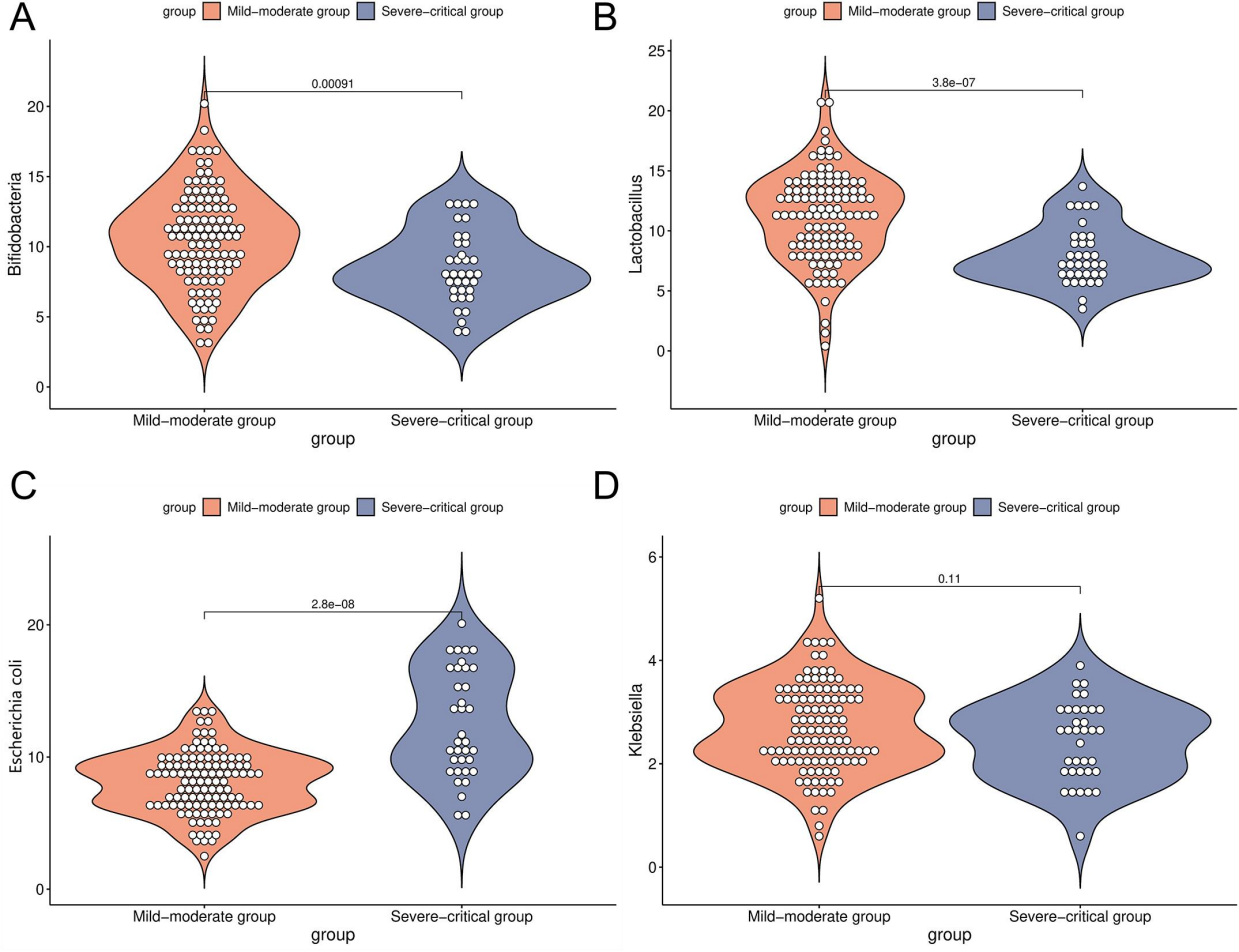
Comparison of intestinal microorganism indicators in the mild-moderate and severe-critical groups [**(A)** was a comparison of Bifidobacteria in the mild-moderate and severe-critical groups; **(B)** was a comparison of Lactobacillus in two groups; **(C)** was a comparison of Escherichia coli in two groups; **(D)** was a comparison of Klebsiella in two groups].

The dysregulation of intestinal microbiota is closely associated with the pathogenesis of asthma. To characterize the intestinal microbial profiles in patients with the mild-moderate asthma compared to those with the severe-critical asthma, the findings of this study revealed no significant difference in the abundance of Klebsiella between the two groups (2.70 ± 0.85 vs. 2.43 ± 0.80) (*P* > 0.05). However, the mild-moderate group exhibited significantly higher levels of Bifidobacteria (10.56 ± 3.42), and Lactobacillus (11.23 ± 3.65) relative to the severe-critical group (8.47 ± 2.62), (7.91 ± 2.43). Conversely, the abundance of Escherichia coli (8.14 ± 2.31) was significantly lower in the mild-moderate group compared to the severe-critical group (12.50 ± 4.06) (*P* < 0.05, Figure 2).

### Comparison of metabolites in the mild-moderate and severe-critical groups

**Figure 3:**
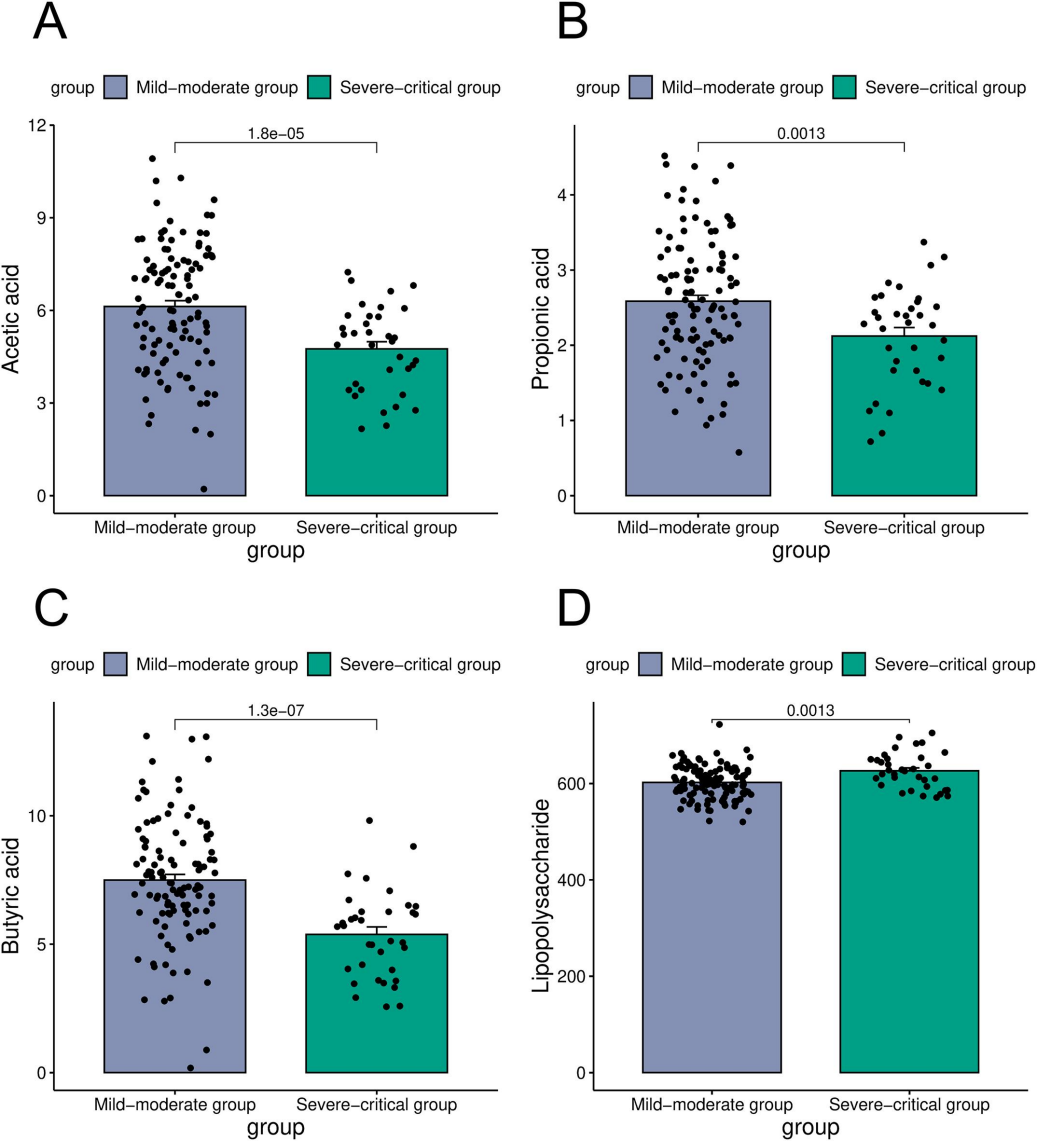
Comparison of metabolites in the mild-moderate and severe-critical groups [**(A)** was a comparison of acetic acid in the mild-moderate and severe-critical groups; **(B)** was a comparison of propionic acid in two groups; **(C)** was a comparison of butyric acid in two groups; **(D)** was a comparison of lipopolysaccharide in two groups).

Intestinal microorganisms metabolize nutrients derived from the host to produce a diverse array of metabolites that play a crucial role in immune regulation. In the present study, an analysis of intestinal microbial metabolites in patients categorized into the mild-moderate and severe-critical groups revealed significant differences. Specifically, the concentration of acetic acid was markedly higher in the mild-moderate group (6.13 ± 2.02 mmol/L) compared to the severe-critical group (4.75 ± 1.38 mmol/L). Similarly, propionic acid levels were elevated in the mild-moderate group relative to the severe-critical group (2.58 ± 0.84 mmol/L vs. 2.12 ± 0.67 mmol/L). Furthermore, butyric acid concentrations were significantly greater in the mild-moderate group (7.49 ± 2.36 mmol/L) than in the severe-critical group (5.38 ± 1.71 mmol/L) In contrast, lipopolysaccharide levels were lower in the mild-moderate group (602.15 ± 34.58 mmol/L) compared to the severe-critical group (676.24 ± 40.15 mmol/L). (*P* < 0.05, Figure 3).

### Relationship between severity of condition and metabolites of intestinal microorganisms in children with allergic asthma

**Figure 4:**
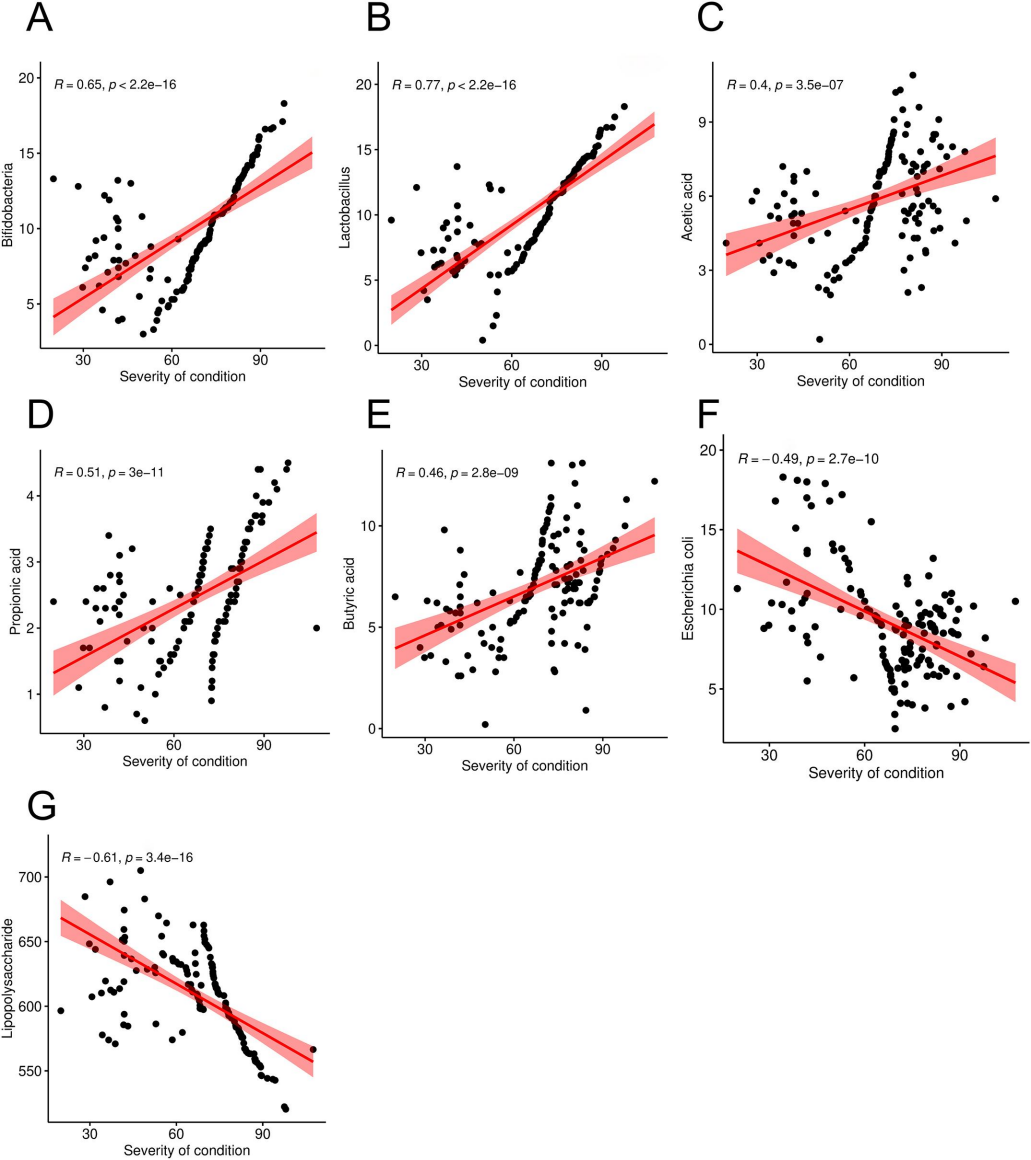
Correlation between severity of condition (PEF level) and intestinal microorganism metabolites in children with allergic asthma [**(A)** was a correlation between PEF and Bifidobacteria; **(B)** was a correlation between PEF and Lactobacillus; **(C)** was a correlation between PEF and Acetic acid; **(D)** was a correlation between PEF and Propionic acid; **(E)** was a correlation between PEF and Butyric acid; **(F)** was a correlation between PEF and Escherichia coli; **(G)** was a correlation between PEF and Lipopolysaccharide].

Correlation scatter plots were constructed to examine the association between disease severity of condition and intestinal microbial metabolites in pediatric patients with allergic asthma. The findings indicated that the PEF in these children was negatively correlated with the abundance of Bifidobacteria, Lactobacillus, acetic acid, propionic acid, and butyric acid (*r* = 0.65, 0.77, 0.40, 0.51, and 0.46, *P* < 0.05), but positive correlation with Escherichia coli and lipopolysaccharide (*r* = −0.49, −0.61, *P* < 0.05 Figure 4).

### The value of intestinal microorganism metabolites in predicting severity of condition in children with allergic asthma

**Figure 5:**
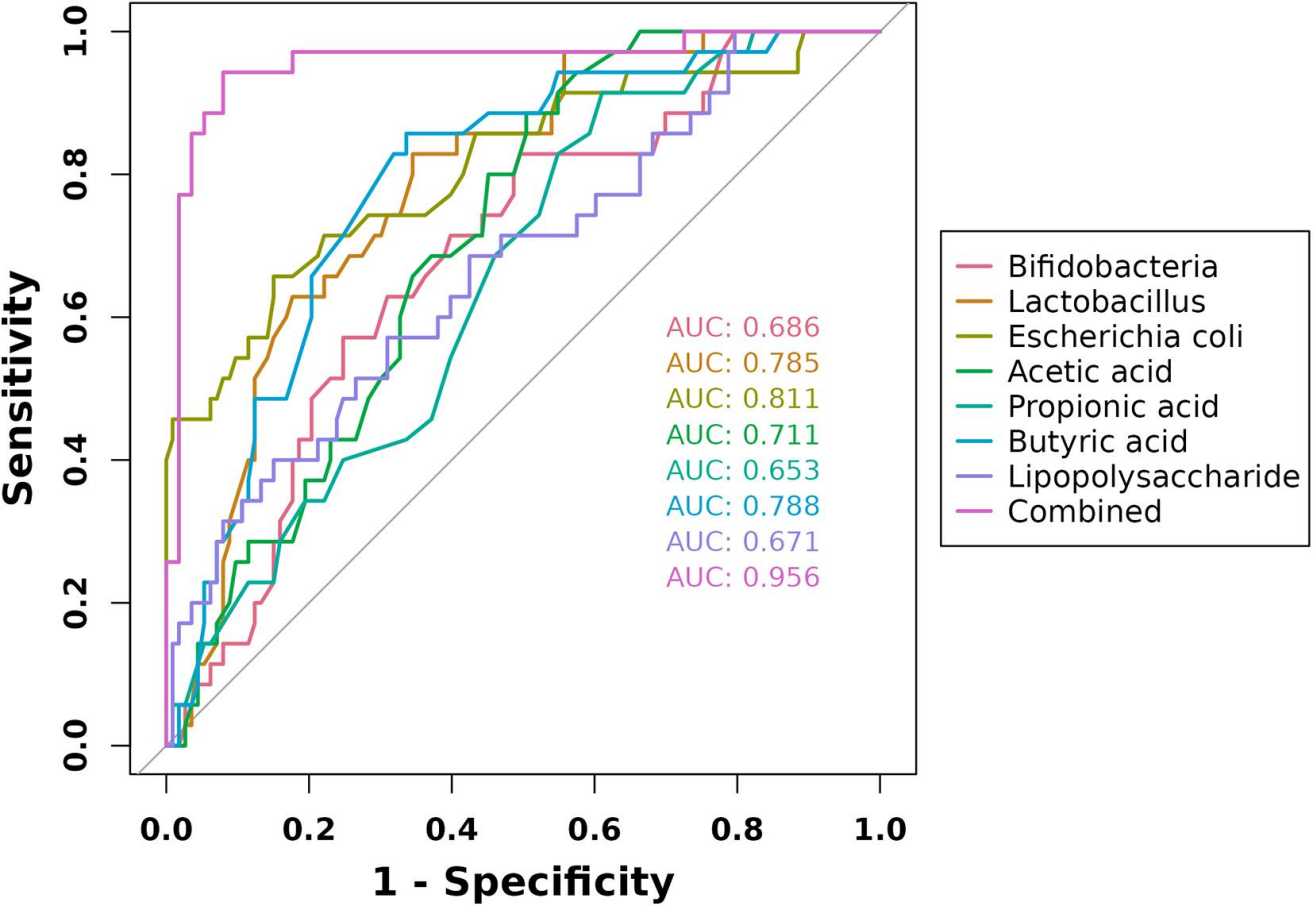
ROC curves of intestinal microorganism metabolites in predicting severity of condition in children with allergic asthma.

ROC curves were generated to evaluate the predictive value of intestinal microbial metabolites for disease severity in pediatric patients with allergic asthma. The analysis revealed that the AUCs for Bifidobacteria, Lactobacillus, Escherichia coli, acetic acid, propionic acid, butyric acid, and lipopolysaccharide in predicting severity of condition were 0.686, 0.785, 0.811, 0.711, 0.653, 0.788, and 0.671, Notably, the combined predictive model yielded an AUC of 0.956, which was higher than that of individual predictors (*P* < 0.05 Figure 5). These findings suggest that metabolites derived from intestinal microorganisms possess substantial potential for accurately predicting disease severity in children with allergic asthma.

## Discussion

Currently, the clinical severity of allergic asthma is categorized into mild, moderate, severe, and critical levels. Children presenting with severe-critical forms exhibit pronounced clinical symptoms and often experience significant airway inflammatory responses. In extreme cases, it may progress to chronic lung disease or pulmonary heart disease, posing substantial risks to pediatric health (14). Nevertheless, the specificity of clinical indicators used to assess the severity of allergic asthma remains highly limited. Consequently, there is an urgent need to identify more precise and reliable biomarkers to support diagnostic and therapeutic decision-making.

Prior research has identified allergic asthma as a multifaceted disorder marked by persistent inflammation and heightened airway responsiveness, with disease severity being correlated with various inflammatory biomarkers (15). Neutrophils, as a critical element of the innate immune system, have been observed at significantly elevated levels in children with asthma relative to their non-asthmatic counterparts. Furthermore, increased neutrophil counts are closely linked to clinical manifestations, including reduced pulmonary function and a higher incidence of acute exacerbations (16). Leukocytes constitute a critical component of the immune system. In pediatric patients experiencing acute asthma exacerbations accompanied by severe symptoms, leukocyte levels tend to be elevated as a result of the body's stress response. CRP, an acute-phase reactant, exhibits a marked increase in concentration during inflammatory and infectious processes. Furthermore, CRP levels progressively rise in correlation with the severity of the condition in children undergoing acute asthma attacks (17, 18). This indicates that neutrophils, leukocytes, and CRP are associated with the severity of allergic asthma, however, they present only certain pathophysiological aspects of the disease and are insufficient for a comprehensive and precise evaluation of its severity. Notably, a retrospective study found no statistically significant difference in CRP levels when comparing mild and moderate-to-severe asthma groups (19). Consistent with the findings of the present study, no statistically significant differences were observed in neutrophil counts, leukocyte levels, and CRP between the mild-moderate and severe-critical groups. This outcome may be attributed to the heterogeneous nature of allergic asthma. Specifically, children with mild-moderate asthma typically exhibit eosinophil-driven inflammatory response, whereas those with severe-critical asthma may present with inflammatory processes involving both neutrophils and eosinophils. Consequently, the neutrophil elevation in the mild-moderate group may not be pronounced. Additionally, the apparent increase of neutrophils within the severe-critical group could have been obscured by other confounding factors. These considerations, combined with the limited sample size of the current study, may have reduced the statistical power to detect significant differences between the two groups (20, 21).

In recent years, advancements in medical technology have led to clinical evidence demonstrating that intestinal microbiota significantly contribute to the pathogenesis and treatment of asthma. These microorganisms primarily affect the development and severity of the disease by modulating immune responses, as well as inflammatory and metabolic pathways (22). Prior research has identified an increased relative abundance of Lactobacillus in individuals with mild-moderate asthma, whereas a decreased relative abundance of Lactobacillus has been observed in those with severe asthma (23). The findings of this study corresponded with clinical observations, indicating that the abundance of Bifidobacteria and Lactobacillus was greater in the mild-moderate group compared to the severe-critical group, whereas Escherichia coli levels were lower in the mild-moderate group relative to the severe-critical group. Notably, Bifidobacteria and Lactobacillus, as intestinal probiotics, play a crucial role in maintaining the equilibrium of the gut microbiota, enhancing intestinal barrier function, suppressing the proliferation of pathogenic bacteria, and consequently mitigating intestinal inflammation and immune dysregulation. According to the findings reported (24), there is a significant negative correlation between the abundance of Bifidobacterium and asthma symptom scores. Furthermore, the role of Bifidobacterium in facilitating intestinal barrier repair has been shown to directly contribute to a reduction in the incidence of airway hyperresponsiveness. A separate investigation (25) suggested that supplementation with Lactobacillus markedly decreases the frequency of asthma exacerbations, implying that Lactobacillus mediates its effects via the gut microbiota-metabolite-lung axis. Consequently, the elevated abundance of Bifidobacteria and Lactobacillus observed in the mild-moderate group suggests their potential role in preserving intestinal microecological stability. Additionally, these microorganisms may contribute to mitigating systemic immune response abnormalities, thereby substantially decreasing the severity of the condition. Escherichia coli is a conditionally pathogenic bacterium residing in the intestinal tract. An imbalance in the intestinal microbiota can lresult in its excessive proliferation, leading to intestinal inflammation. This inflammatory state influences pulmonary immune responses via the “gut-lung axis” in pediatric populations, thereby significantly exacerbating the clinical condition (26). Consequently, there exists a strong association between elevated Escherichia coli levels observed in the severe-critical group and both dysbiosis of the intestinal flora and aberrant immune responses.

Beyond the intestinal microbiota themselves, their metabolic byproducts have been implicated in the severity of allergic asthma. Research findings indicate that individuals with mild asthma exhibit the highest concentrations of acetic acid and butyric acid, with intermediate levels observed in the moderate group, and the lowest levels detected in those with severe asthma (27). The findings of this study were consistent with previous research, demonstrating that the concentrations of acetic acid, propionic acid and butyric acid were significantly elevated in the mild-moderate group compared to the severe-critical group. Conversely, levels of lipopolysaccharide were lower in the mild-moderate group relative to the severe-critical group.These results suggest that metabolites derived from intestinal microbiota are critically involved in modulating the severity of allergic asthma. Specifically, acetic acid, propionic acid, and butyric acid are short-chain fatty acids with distinct immunomodulatory functions. Acetic acid can activate GPR41 and GPR43 receptors, facilitating calcium ion influx and thereby influencing immune cell function. Additionally, acetic acid inhibits histone deacetylase activity, contributing to the regulation of immune responses. Propionic acid similarly suppresses histone deacetylase activity, promoting the differentiation and proliferation of regulatory T-cells (Tregs), which play a pivotal role in maintaining immune homeostasis. Butyric acid not only modulates Treg3 cells, but also inhibits histone deacetylase activity, and enhances the activity of transcription factors. These combined effects significantly improve immune tolerance and contribute to the amelioration of airway responsiveness in children with allergic asthma (28, 29). Moreover, lipopolysaccharide constitutes a principal component of the cell wall in gram-negative bacteria, and functions as an endotoxin. It has the capacity to activate the immune system and induce systemic inflammation, thereby contributing to the exacerbation of asthma. Consequently, the intestinal barrier function of children with milder forms of the disease remains relatively preserved, effectively inhibiting the absorption and translocation of lipopolysaccharide. In contrast, children with severe disease exhibit compromised intestinal barrier integrity, which facilitates the translocation of lipopolysaccharide into the systemic circulation (30).

This study employed correlation and ROC curve analyses to investigate the relationship between disease severity in children and specific intestinal microorganisms and their metabolites. The findings revealed a negative correlation between disease severity and the presence of Bifidobacteria, Lactobacillus, acetic acid, propionic acid and butyric acid. Conversely, a positive correlation was observed with Escherichia coli and lipopolysaccharides. Furthermore, intestinal microbiota and their metabolic products demonstrated significant predictive value for assessing the severity of atopic asthma in pediatric patients. These results suggest that modulation of the gut microbiota and its metabolites may serve as a valuable reference basis for the clinical prevention and management of allergic asthma. 16s RNA sequencing is a methodological approach employed to analyze the diversity of intestinal microbiota, primarily utilized for the identification and classification of bacterial species. This technique facilitates the assessment of the composition and dynamic alterations within the intestinal flora. Recent studies have demonstrated its clinical utility in enabling more precise evaluation of disease severity and the development of individualized treatment strategies for pediatric patients with allergic asthma by detecting intestinal microorganisms and their associated metabolites (31, 32). Bifidobacteria and Lactobacillus, as key probiotic constituents of the intestinal microbiota, play a critical role in maintaining intestinal barrier integrity. A decline in their populations can compromise this barrier function, facilitating the translocation of pathogenic bacteria and their metabolites into the systemic circulation, thereby exacerbating disease severity. Additionally, decreased concentrations of short-chain fatty acids such as propionic acid and butyric acid may impair the suppression of inflammatory responses, further intensifying the pathological condition. Concurrently, levels of Escherichia coli and lipopolysaccharides contribute to heightened intestinal inflammation and disruption of barrier function, promoting the entry of harmful substances into the bloodstream and aggravating the clinical severity in pediatric patients (33).

This study presents a novel integration of intestinal microorganisms and their metabolites, employing correlation and ROC curve analyses to validate the relationship between the combined biomarker panel and disease severity, as well as its predictive utility for disease progression. These findings establish a foundational basis for the future clinical development of the diagnostic algorithms. Furthermore, ongoing advancements in biotechnology and artificial intelligence methodologies are enhancing the maturity of detection technologies for intestinal microorganisms and their metabolites, thereby offering substantial technical support for the development of diagnostic algorithms. However, this study exhibits certain limitations. Firstly, the relatively small sample size may constrain the generalizability of the findings to broader populations. Secondly, intestinal metabolites are produced through the combined influence of the host, microbial communities, and dietary intake, complicating the precise attribution of their origins. Lastly, while metabolites hold promise as potential biomarkers, further investigation is required to effectively translate these findings into practical clinical diagnostic applications. To address this issue, future clinical research should incorporate multi-regional and multi-ethnic pediatric cohorts, implement standardized protocols for sample collection and analysis, and validate the generalizability of metabolic biomarkers. Additionally, longitudinal studies are warranted to monitor the dynamic alterations in the integrated microbiota and metabolite profiles of children with asthma. Furthermore, interdisciplinary approaches combining immunology and environmental science are essential to elucidate the interactions among intestinal microbial metabolites, host immune responses, and environmental exposures.

In summary, the composition of intestinal microbiota and their associated metabolites exhibits abnormal expression patterns in pediatric patients with allergic asthma. Furthermore, these alterations correlate with disease severity, suggesting their potential utility as significant biomarkers predicting the clinical progression of allergic asthma in children.

## Data Availability

The original contributions presented in the study are included in the article/Supplementary Material, further inquiries can be directed to the corresponding authors.

## References

[1] Abdel-Aziz MI Hashimoto S Neerincx AH Haarman EG Cecil A Lintelmann J Metabotypes are linked to uncontrolled childhood asthma, gut microbiota, and systemic inflammation. J Allergy Clin Immunol. (2025) 156:339–51. 10.1016/j.jaci.2025.04.017 40280190

[2] Bhutta NK Xu X Jian C Wang Y Liu Y Sun J Gut microbiota mediated T cells regulation and autoimmune diseases. Front Microbiol. (2024) 15:1477187. 10.3389/fmicb.2024.1477187 39749132 PMC11694513

[3] Cheng ZX Wu YX Jie ZJ Li XJ Zhang J. Genetic evidence on the causality between gut microbiota and various asthma phenotypes: a two-sample mendelian randomization study. Front Cell Infect Microbiol. (2023) 13:1270067. 10.3389/fcimb.2023.1270067 38274730 PMC10808785

[4] Chiu CY Chang KC Chang LC Wang CJ Chung WH Hsieh WP Phenotype-specific signatures of systems-level gut microbiome associated with childhood airway allergies. Pediatr Allergy Immunol. (2023) 34:e13905. 10.1111/pai.13905 36705037

[5] Chiu CY Chiang MH Kuo CN Cheng ML Lin G. Multi-biofluid metabolomics analysis of allergic respiratory rhinitis and asthma in early childhood. World Allergy Organ J. (2025) 18:101013. 10.1016/j.waojou.2024.101013 39810829 PMC11731466

[6] Cordova S Tena-Garitaonaindia M Alvarez-Mercado AI Gamez-Belmonte R Gomez-Llorente MA Sanchez de Medina F Differential modulation of mouse intestinal organoids with fecal luminal factors from obese, allergic, asthmatic children. Int J Mol Sci. (2024) 25:866. 10.3390/ijms25020866 38255939 PMC10815115

[7] Depner M Taft DH Peschel S Roduit C Karvonen AM Barnig C The janus face of bifidobacterium in the development of atopic eczema: a role for compositional maturation. Pediatr Allergy Immunol. (2025) 36:e70041. 10.1111/pai.70041 39932047 PMC11812080

[8] Dera N Kosinska-Kaczynska K Zeber-Lubecka N Brawura-Biskupski-Samaha R Massalska D Szymusik I Impact of early-life microbiota on immune system development and allergic disorders. Biomedicines. (2025) 13:121. 10.3390/biomedicines13010121 39857705 PMC11762082

[9] Fadlyana E Soemarko DS Endaryanto A Haryanto B Darma A Dewi DK The impact of air pollution on gut microbiota and children’s health: an expert consensus. Children (Basel). (2022) 9:765. 10.3390/children9060765 35740702 PMC9222189

[10] Fiocchi A Cabana MD Mennini M. Current use of probiotics and prebiotics in allergy. J Allergy Clin Immunol Pract. (2022) 10:2219–42. 10.1016/j.jaip.2022.06.038 35792336

[11] Hoskinson C Dai DL Del Bel KL Becker AB Moraes TJ Mandhane PJ Delayed gut microbiota maturation in the first year of life is a hallmark of pediatric allergic disease. Nat Commun. (2023) 14:4785. 10.1038/s41467-023-40336-4 37644001 PMC10465508

[12] Iliodromiti Z Triantafyllou AR Tsaousi M Pouliakis A Petropoulou C Sokou R Gut microbiome and neurodevelopmental disorders: a link yet to be disclosed. Microorganisms. (2023) 11:487. 10.3390/microorganisms11020487 36838452 PMC9964594

[13] Jin Q Ren F Dai D Sun N Qian Y Song P. The causality between intestinal flora and allergic diseases: insights from a bi-directional two-sample Mendelian randomization analysis. Front Immunol. (2023) 14:1121273. 10.3389/fimmu.2023.1121273 36969260 PMC10033526

[14] Kallio S Jian C Korpela K Kukkonen AK Salonen A Savilahti E Early-life gut microbiota associates with allergic rhinitis during 13-year follow-up in a Finnish probiotic intervention cohort. Microbiol Spectr. (2024) 12:e0413523. 10.1128/spectrum.04135-23 38687061 PMC11324021

[15] Kielenniva K Ainonen S Vanni P Paalanne N Renko M Salo J Microbiota of the first-pass meconium and subsequent atopic and allergic disorders in children. Clin Exp Allergy. (2022) 52:684–96. 10.1111/cea.14117 35212058 PMC9314137

[16] Kim JH Lee SH Kang MJ Hwang SG Park YM Kim BS Host-microbial interactions between PTGR2 and Bifidobacterium in the early life gut of atopic dermatitis children. Pediatr Allergy Immunol. (2022) 33:e13724. 10.1111/pai.13724 34936126

[17] Li J Shi Y Hu L He W Li Y. Assessing gut microbiome alterations in children with allergic rhinitis: associations with allergen-specific IgE levels and sensitization patterns. J Asthma Allergy. (2025) 18:269–81. 10.2147/JAA.S496477 40007795 PMC11853164

[18] Li M Li N Dong Y Zhang H Bai Z Zhang R Soil intake modifies the gut microbiota and alleviates Th2-type immune response in an ovalbumin-induced asthma mouse model. World Allergy Organ J. (2024) 17:100897. 10.1016/j.waojou.2024.100897 38655570 PMC11035114

[19] Li Q Fan Y Luo R Hu J Wang L Ai T. Related risk factors that predict moderate to severe asthma attack in children: analysis based on logistic regression and decision tree. Int J Gen Med. (2025) 18:3919–31. 10.2147/IJGM.S530736 40688461 PMC12275991

[20] Li Q Shen Y Guo X Xu Y Mao Y Wu Y Betanin dose-dependently ameliorates allergic airway inflammation by attenuating Th2 response and upregulating cAMP-PKA-CREB pathway in asthmatic mice. J Agric Food Chem. (2022) 70:3708–18. 10.1021/acs.jafc.2c00205 35298142

[21] Meng CY Gong XL Zhao R Lu Q Dong XY. Effect of maternal exposure to lipopolysaccharide during pregnancy on allergic asthma in offspring in mice. Zhonghua Er Ke Za Zhi. (2022) 60:302–6. 10.3760/cma.j.cn112140-20220130-00100 35385934

[22] Lv H Wang Y Gao Z Liu P Qin D Hua Q Knowledge mapping of the links between the microbiota and allergic diseases: a bibliometric analysis (2002–2021). Front Immunol. (2022) 13:1045795. 10.3389/fimmu.2022.1045795 36389800 PMC9650552

[23] Menegati LM de Oliveira EE Oliveira BC Macedo GC de Castro ESFM. Asthma, obesity, and microbiota: a complex immunological interaction. Immunol Lett. (2023) 255:10–20. 10.1016/j.imlet.2023.01.004 36646290

[24] Lv J Zhang Y Liu S Wang R Zhao J. Gut-lung axis in allergic asthma: microbiota-driven immune dysregulation and therapeutic strategies. Front Pharmacol. (2025) 16:1617546. 10.3389/fphar.2025.1617546 40822476 PMC12350297

[25] Chen PC Hsu HY Liao YC Lee CC Hsieh MH Kuo WS Oral administration of Lactobacillus delbrueckii subsp. lactis LDL557 attenuates airway inflammation and changes the gut microbiota in a Der p-sensitized mouse model of allergic asthma. Asian Pac J Allergy Immunol. (2024). 10.12932/AP-200823-1672 38710644

[26] Mousavian AH Zare Garizi F Ghoreshi B Ketabi S Eslami S Ejtahed HS The association of infant and mother gut microbiomes with development of allergic diseases in children: a systematic review. J Asthma. (2024) 61:1121–35. 10.1080/02770903.2024.2332921 38506489

[27] Saeed NK Al-Beltagi M Bediwy AS El-Sawaf Y Toema O. Gut microbiota in various childhood disorders: implication and indications. World J Gastroenterol. (2022) 28:1875–901. 10.3748/wjg.v28.i18.1875 35664966 PMC9150060

[28] Sasaki M Suaini NHA Afghani J Heye KN O'Mahony L Venter C Systematic review of the association between short-chain fatty acids and allergic diseases. Allergy. (2024) 79:1789–811. 10.1111/all.16065 38391245

[29] Suarez-Martinez C Santaella-Pascual M Yague-Guirao G Garcia-Marcos L Ros G Martinez-Gracia C. The early appearance of asthma and its relationship with gut Microbiota: a narrative review. Microorganisms. (2024) 12:1471. 10.3390/microorganisms12071471 39065238 PMC11278858

[30] Wan J Song J Lv Q Zhang H Xiang Q Dai H Alterations in the gut microbiome of young children with airway allergic disease revealed by next-generation sequencing. J Asthma Allergy. (2023) 16:961–72. 10.2147/JAA.S422537 37700874 PMC10494927

[31] Wilson NG Hernandez-Leyva A Schwartz DJ Bacharier LB Kau AL. The gut metagenome harbors metabolic and antibiotic resistance signatures of moderate-to-severe asthma. FEMS Microbes. (2024) 5:xtae010. 10.1093/femsmc/xtae010 38560624 PMC10981462

[32] Zheng J Huang Y Zhang L Liu T Zou Y He L Role of the gut-lung microbiome axis in airway inflammation in OVA-challenged mice and the effect of azithromycin. J Inflamm Res. (2025) 18:2661–76. 10.2147/JIR.S506688 40008084 PMC11853874

[33] Yang L Lin Z Gao T Wang P Wang G. The role of skin-gut-lung microbiome in allergic diseases. J Allergy Clin Immunol Pract. (2025) 13:1935–1942.e4. 10.1016/j.jaip.2025.04.041 40320144

